# Dihydroartemisinin Reduces Irradiation-Induced Mitophagy and Radioresistance in Lung Cancer A549 Cells via CIRBP Inhibition

**DOI:** 10.3390/life12081129

**Published:** 2022-07-27

**Authors:** Shunlong Wu, Zhaodong Li, Haiyu Li, Kui Liao

**Affiliations:** 1 Department of Oncology, The First Affiliated Hospital of Chongqing Medical University, Chongqing 400016, China; 203058@hospital.cqmu.edu.cn; 2 Basic Medical College, Chongqing Medical University, Chongqing 400016, China; 190570@cqmu.edu.cn; 3 Chongqing Research Institute, Chinese Academy of Sciences, Chongqing 400016, China; lihaiyu@cqmu.edu.cn

**Keywords:** autophagy, radiotherapy, dihydroartemisinin, fractional radiation

## Abstract

Radiotherapy is a major therapeutic strategy for lung cancer, and radiation resistance (radioresistance) is an important cause of residual and recurring cancer after treatment. However, the mechanism of radioresistance remains unclear. Mitochondrial autophagy (mitophagy), an important selective autophagy, plays an important role in maintaining cell homeostasis and affects the response to therapy. Recent studies have shown that dihydroartemisinin (DHA), a derivative of artemisinin, can increase the sensitivity to treatment in multiple types of cancer, including lung cancer. The purpose of this study was to elucidate the function and molecular mechanisms of DHA-regulating mitophagy and DHA-reducing radioresistance in lung cancer A549 cells. We first constructed the radioresistant lung cancer A549 cells model (A549R) through fractional radiation, then elucidated the function and mechanism of DHA-regulating mitophagy to reduce the radioresistance of lung cancer by genomic, proteomic, and bioinformatic methods. The results showed that fractional radiation can significantly induce radioresistance and mitophagy in A549 cells, DHA can reduce mitophagy and radioresistance, and the inhibition of mitophagy can reduce radioresistance. Protein chip assay and bioinformatics analysis showed the following: Cold-Inducible RNA Binding Protein (CIRBP) might be a potential target of DHA-regulating mitophagy; CIRBP is highly expressed in A549R cells; the knockdown of CIRBP increases the effect of DHA, reduces mitophagy and radioresistance, and inhibits the mitophagy-related PINK1/Parkin pathway. In conclusion, we believe that DHA reduces radiation-induced mitophagy and radioresistance of lung cancer A549 cells via CIRBP inhibition.

## 1. Introduction

Lung cancer is the most common malignant tumor and the main cause of cancer-related death. According to statistics, 1.6 million people die of lung cancer every year [1]. Radiotherapy is the only treatment strategy that can be used in all stages of the disease. Approximately 77% of lung cancer patients need radiotherapy [2]. On the whole, radiotherapy can improve the five-year local control rate by 8.3% and the survival rate by 4% [3]. Therefore, radiotherapy has an important impact on the local control and survival rates of patients.

With the development of medical imaging and radiotherapy technology, radiotherapy has experienced the process from two- and three-dimensional conformal radiotherapy to intensity modulated radiation therapy (IMRT). Coupled with the application of auxiliary technologies such as image-guided radiation therapy (IGRT), respiratory gating, adaptive radiotherapy, and proton radiotherapy, the local control rate of lung cancer has been greatly improved [2]. However, there are still some patients with residual, final recurrence or metastasis after radiotherapy due to the resistance of tumors to treatment. There is evidence that only 26% of all patients with non-small cell lung cancer (NSCLC) are alive five years after diagnosis [4]. The mechanisms of radioresistance are complex, related to the mode of radiation dose fractional, cell cycle, oxygenation level, autophagy, and so on [5]. Therefore, elucidating the molecular mechanism of the radioresistance of lung cancer and finding ideal strategies or drugs to increase sensitivity to radiation are of great significance to improve the local control rate of lung cancer and reduce recurrence and metastasis.

Artemisinin is a well-known antimalarial drug, and dihydroartemisinin (DHA) is a derivative of artemisinin. Possessing anticancer activity and low toxicity, DHA is being used in basic and clinical research as an anticancer drug or a therapeutic sensitizer [6]. However, the mechanism of DHA reduce the sensitivity to therapeutic (including radiotherapy) remains unclear. A recent study has shown that DHA can eliminate the radiation resistance of non-small cell lung cancer through abrogating immunity escaping and promoting radiation sensitivity by inhibiting PD-L1 expression [7]. In addition, our previous study found that DHA inhibits proliferation and induces apoptosis via AKT/GSK3β/cyclinD1 pathway inhibition in A549 lung cancer cells. Therefore, DHA plays an important role in lung cancer biological behavior and lung cancer response to treatment [8].

Autophagy is a basic process of the catabolism of cellular components. The biological importance and molecular mechanisms of autophagy have been extensively studied. Mitochondria are essential organelles for energy production and cell survival. Mitophagy is an important selective autophagy, which plays an important role in the self-renewal of mitochondria and maintenance of cell functions. Several studies have suggested that mitophagy contributes to tumor growth and radioresistance. For example, mitophagy induced by reactive oxygen species (ROS) can initiate the sensitization of cancer cells to IR in the breast cancer cell line MCF-7 and the cervical cancer cell line HeLa [9]. LACTB2 renders radioresistance by activating mitophagy in nasopharyngeal carcinoma [10]. Putative protein kinase 1 (PINK1) and Parkinson disease protein 2 (Parkin) are related to mitophagy [9,10,11], but the mechanism of PINK1-dependent mitophagy in radioresistance remains unclear.

In this study, the radiation-induced radioresistant lung cancer A549 cell model was first constructed by fractional radiation, and then, cloning assay, confocal imaging, protein chip, gene knockdown, flow cytometry, RT-PCR, Western blot, and bioinformatics methods were used to explore the function and mechanisms of DHA in regulating mitophagy and reducing the radioresistance of lung cancer.

## 2. Materials and Methods

### 2.1. Cell Lines, Reagents

Human lung cancer cell line A549 was obtained from Chongqing Medical University (Chongqing, China) and cultured in Dulbecco’s Modified Eagle’s medium (DMEM; Gibco, Grand Island, NY, USA), supplemented with 10% fetal bovine serum (MRC, Jintan, China) and 1% penicillin/streptomycin (Invitrogen, Carlsbad, CA, USA).

Hydroxychloroquine and Mdivi-1 were purchased from Sigma Aldrich (Shanghai, China) Co., Ltd. Dihydroartemisinin (DHA) was purchased from Dibai Chemical Co., Ltd. (Hubei, China).

### 2.2. Radiation-Induced Radioresistance of Lung Cancer A549 Cells

The brand of linac was Varian vital beam of Varian Medical Systems Inc. (Palo Alto, CA, USA).

When the cell confluence reached 80%, a total radiation dosage 40 Gy of X-ray (2 Gy/day, 20 days continuous, dose rate of 4 Gy/min, source–surface distance (SSD) of 100 cm) was delivered. Different analysis was conducted after 24 h from the final irradiation.

### 2.3. Clonogenic Assay

The radiosensitivity of the cells was evaluated using a clonogenic assay. Briefly, cells treated with or without Mdivi-1 or DHA were cultured in six-well plates with a number from 400 to 4000, which were then were irradiated with an individual dose of 0, 2, 4, or 8 Gy. After being cultured for 10–12 days, the colonies were fixed and stained. Colonies containing at least 50 cells were counted using Image J. Plating efficiency was defined as the number of counted colonies divided by the number of seeded cells, and the surviving fraction at each dose was defined as the number of counted colonies divided by the number of seeded cells times the plating efficiency. The link between survival fractions and irradiation dose was mainly described via the multitarget–single hitting model of GraphPad Prism 8. The sensitization enhancement ratio (SER) is defined as the ratio of the dose of radiation alone to that of radiation combined with the sensitizer required to achieve the same biological effect, and the SER were obtained quantitatively from the curves of the multitarget–single hitting model.

### 2.4. Real-Time Polymerase Chain Reaction (RT-PCR)

To quantify gene expression, RT-PCR was performed following the instructions of the SYBR^®^ Premix Ex TaqTM II (Tli RNaseH Plus) kit of Takara Biotechnology Co., Ltd. (Dalian, China). In brief, Trizol was used to extract total RNA, and a reverse transcription kit (Takara) was used to synthesize the cDNA according to the manufacturer’s instructions. Then, PCR was performed. The expression levels of the target genes relative to actin were determined using a SYBR Green-based comparative CT method (relative fold-change, 2^−ΔΔCt^). The primers were designed by Sangon Biotech Co., Ltd. (Shanghai, China), and are as follows:
**Gene Name**
**Sequence (5′ → 3′)**CIRBPForwardAGGGCTGAGTTTTGACACCAAReverseACAAACCCAAATCCCCGAGATPINK1ForwardGCCTCATCGAGGAAAAACAGGReverseGTCTCGTGTCCAACGGGTCActinForwardGCACCACACCTTCTACAATGAReverseGTCATCTTCTCGCGGTTGGC

### 2.5. Western Blot

Anti-β-actin, anti-microtubule-associated protein 1 light chain 3 (LC3), anti-PINK1, and anti-Parkin were purchased from Cell Signaling Technology (Danvers, MA, USA). A protein extraction kit and a mitochondria isolation kit were purchased from Keygenbiotech Co., Ltd. (Nanjing, China).

The extraction of mitochondrial protein was performed according to Cat Number KGP850/KGP8100 instructions from Keygenbiotech Co., Ltd. (Nanjing, China). The membranes were incubated with one or more of the following primary antibodies: anti-actin, anti-PINK1, anti-Parkin, anti-LC3 (each 1:1000), and secondary antibody (1:2000). The band intensities were detected using a Western blot analysis system. The proteins were normalized to actin and quantified using ChemiDoc™XRS. Three independent experiments were performed.

### 2.6. Protein Chip

We linked biotin to DHA to form a biotin–DHA-labeled probe, which was then incubated with the protein chip. After washing, the specific binding sites (i.e., targets) between the drug and the protein fixed on the chip were determined according to the combination of the biotin-labeled probe on the detection protein chip and BODIPY-FL streptomyclophilin, and then the proteins at these binding sites were analyzed by computer software. The protein chips were constructed by Wayen Biotechnologies (Shanghai) Co., Ltd. (Shanghai, China).

### 2.7. Gene Knockdown

Lentiviral human CIRBP-targeting short hairpin RNA (shRNA) was purchased from Shanghai GenePharma Co., Ltd. (Shanghai, China). Knockdown of CIRBP was performed conventionally according to the method we reported previously [12]. The sequence shCIRBP: 5′-CATGAATGGGAAGTCTGTA-3′ and 5′-TCTCAAAGTACGGACAGAT-3′ and the negative control: 5′-TTCTCCGAACGTGTCACGT-3′ were used as RNA interference and negative control, respectively. The cells were grown to the log phase in T25, dispersed into single cells and seeded 1 × 10^5^/well into a 24-well plate. After 24 h, the cells were transfected with the virus shRNA or a virus non-carrier in SFM. The gene and protein expression levels of CIRBP were evaluated by RT-PCR and Western blot analysis, respectively.

### 2.8. Immunofluorescence and Confocal Imaging

A Mito-Tracker Green dye kit and Lyso-Tracker Green dye were purchased from Beyotime^®^ Biotechnology (Hangzhou, China).

Confocal imaging: We labeled cell mitochondria and lysosomes with Mito-Tracker Red (1:9000) and Lyso-Tracker Green (1:9000), respectively. After incubation at 37 °C for 1 h, they were washed with PBS three times. Autophagy was observed with a confocal microscope. During autophagy, green lysosomes fuse with red mitochondria to produce yellow fluorescence; the relative fluorescence spot numbers per cell were counted, and a total of 100 cells were counted for each analysis.

Immunofluorescence: PINK1 distribution: After the samples were prepared, the cells’ mitochondria were labeled with Mito-Tracker Red CMXRos (1:9000), then fixed by paraformaldehyde for 20 min, made transparent by Triton X-100 10 min, and sealed by QuickBlock™ Blocking Buffer for 20 min; then, the first antibody PINK1 was added. The second antibody (green fluorescent goat anti-rabbit) was incubated the next day, and then the distribution of PINK1 was observed by fluorescence microscopy.

### 2.9. Flow Cytometry

The treated cells were washed with PBS, to which DCFH-DA with a diluted concentration of 1:1000 and serum-free medium were added and incubated in dark for 30 min. They were then washed with PBS, digested and centrifuged, and then transferred to an EP tube to detect ROS fluorescence by flow cytometry.

### 2.10. Statistics

All experiments were repeated at least three times. Firstly, the normality and variance homogeneity of data were evaluated; if the variance homogeneity was uniform, the differences between groups were analyzed by Student’s *t*-tests, and the differences among the three groups were analyzed using one-way ANOVA, and differences between any two groups were analyzed using least significant difference (LSD) tests. If the variance homogeneity was not uniform, the non-parametric tests were used. All statistical analyses were performed using SPSS version 22.0 for Windows. A *p*-value of <0.05 was considered as statistically significant in all experiments.

## 3. Results

### 3.1. Mitophagy and Radioresistance Were Induced by Fraction Radiation in Lung Cancer A549 Cells

**Figure 1:**
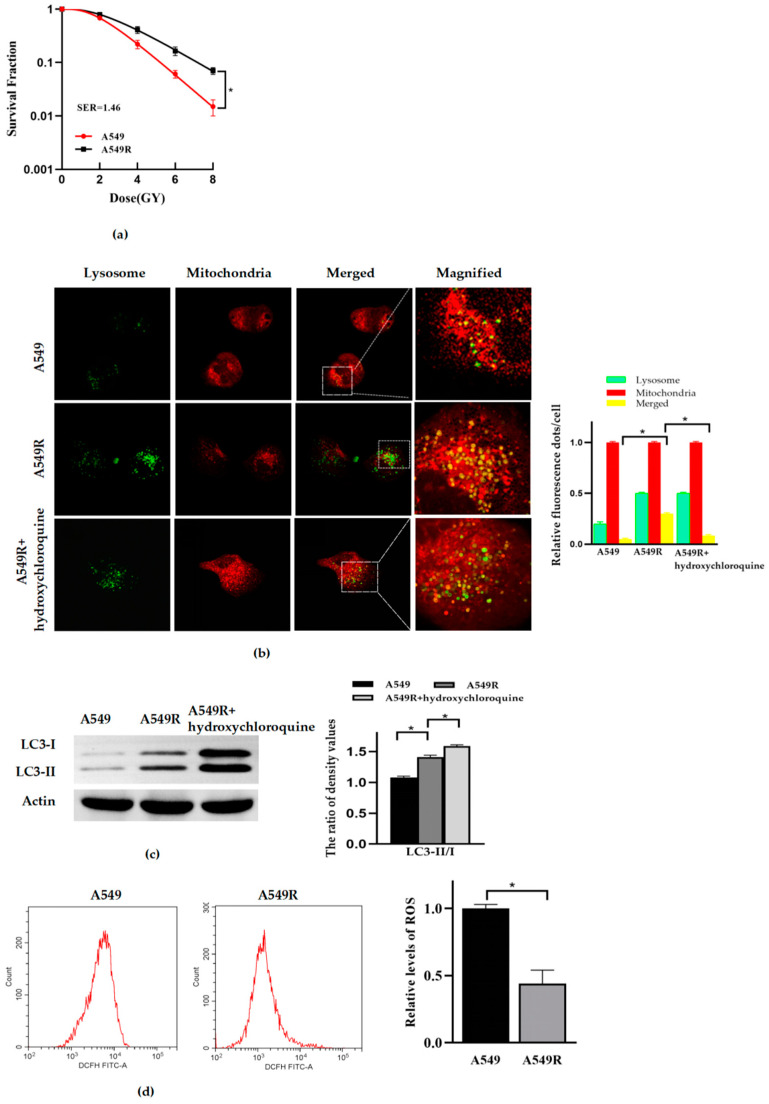
Mitophagy and radioresistance were induced by fractionated radiation in lung cancer A549 cells. (**a**) The survival fractions of cells was fitted by multitarget–single hitting model according to the clonogenic assay. (**b**) Representative fluorescence images of A549 and A549R cells with or without hydroxychloroquine (50 μM, 24 h), after treated with Mito-Tracker (Red) and Lyso-Tracker (Green) (×40) (**Left**). The relative fluorescence spot numbers per cell (**right**). A total of 100 cells were counted for each analysis. (**c**) The expression of proteins LC3-I, LC3-II and actin (**left**), and the relative ratio of LC3-II/LC3-I in A549 and A549R cells treated with or without hydroxychloroquine (50 μM, 24 h) (**right**). (**d**) Relative level of intracellular ROS in A549, A549R cells. * *p* < 0.05 between indicated groups.

First, we constructed the radioresistant lung cancer A549 cells line (A549R) by fractionated radiation with a dosage of 2 Gy X-ray per day, consecutively for 20 days. A colony formation assay was used to identify the radiosensitivity of the cells. Hydroxychloroquine, a late-stage inhibitor of autophagy, can inhibit autophagic flux. It has been widely used to identify whether the increase in autophagy phenomenon is due to an increase in autophagy activation or a blockade of autophagy flux. The level of mitophagy was detected by confocal imaging and Western blot. The level of ROS was detected by flow cytometry. The results showed that the A549R cells had stronger radioresistance than the A549 cells (Figure 1a). Confocal imaging (Figure 1b) and Western blot analysis (Figure 1c) showed that more fusion between mitochondrial and lysosomal occurred, and the expression of protein LC3II and the ratio of LC3II/I were elevated in the A549R cells, compared to that of the A549 cells. Meanwhile, in the presence of hydroxychloroquine (50 μM, 24 h), the fusion between lysosome and mitochondria was blocked, and the accumulation of protein LC3II increased. Taking all of that evidence together, the mitophagy of the A549R cells was significantly enhanced, compared to that of the A549 cells. However, interestingly, the ROS of A549R decreased significantly (Figure 1d).

### 3.2. DHA Reduces Radioresistance and Mitophagy in A549R Cells

**Figure 2:**
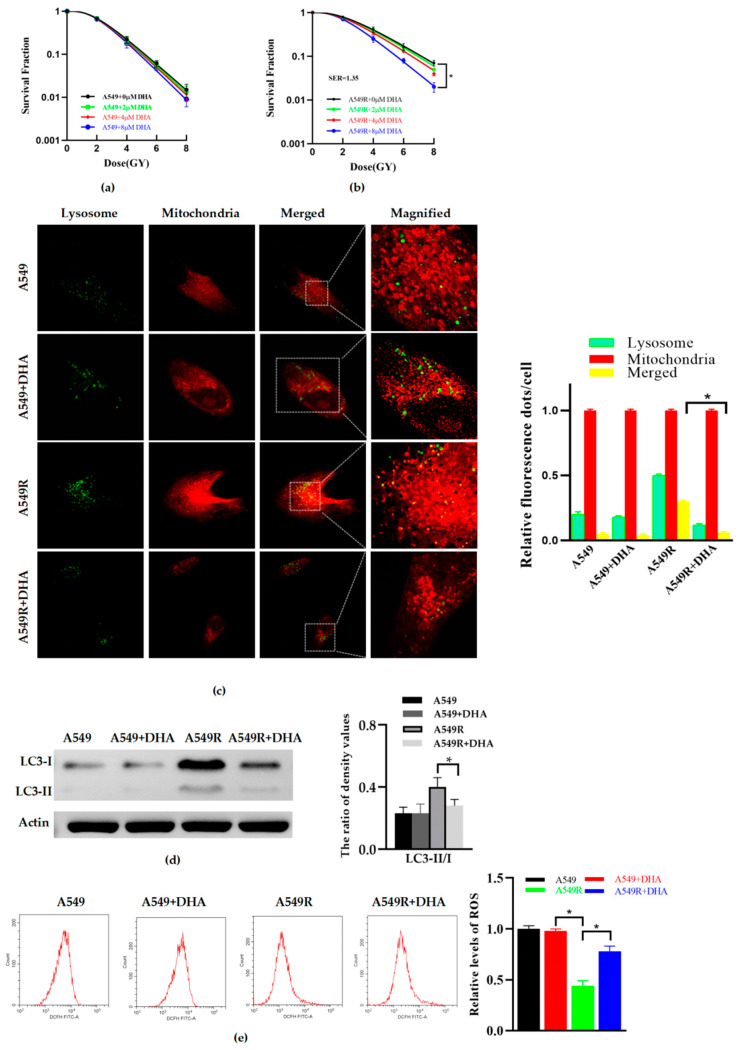
DHA reduces radioresistance and mitophagy in A549R cells. (**a**,**b**) The survival fractions of A549 and A549R cells, treated with DHA (0–8 μM, 24 h), respectively, were fitted by the multitarget–single hitting model according to a clonogenic assay. (**c**) Representative fluorescence images of A549 and A549R cells, treated with or without DHA (8 μM, 24 h), and mitochondria and lysosomes of all that were labeled with Mito-Tracker (red) and Lyso-Tracker (green) (×40) (**left**). Relative fluorescence spot numbers per cell. A total of 100 cells were counted for each analysis (**right**). (**d**) Western blot assay of LC3-I, LC3-II, and actin proteins and the relative ratio of LC3-II/I in A549 and A549R cells treated with or without DHA (8 μM, 24 h). (**e**) The representative images and relative level of ROS in A549 and A549R cells treated with or without DHA (8 μM, 24 h). * *p* < 0.05 between the indicated groups.

A549 and A549R cells were treated with DHA for 24 h, and the results indicated that with an increasing concentration from 0 to 8 μM, the radiation sensitivity of A549 underwent no significant changes (Figure 2a). However, the radioresistance of the A549R cells treated with 8 μM of DHA could be significantly reduced (Figure 2b). In order to explore the effect of DHA on mitophagy, confocal imaging and Western blot were used. Similarly, according to confocal imaging (Figure 2c) and Western blot analysis (Figure 2d), after treatment with 8 μM of DHA for 24 h, the mitophagy was not significantly affected in the A549 cells, but dramatically decreased in the A549R cells. A greater amount of ROS were also produced in the A549R group after treatment with DHA for 24 h at a concentration of 8 μM (Figure 2e). No significant changes were observed in the other groups. To sum up, these data indicate that DHA can reduce the mitophagy and radioresistance of A549R and promote ROS production.

### 3.3. Inhibition of Mitophagy Reduces the Radioresistance of A549R Cells

**Figure 3:**
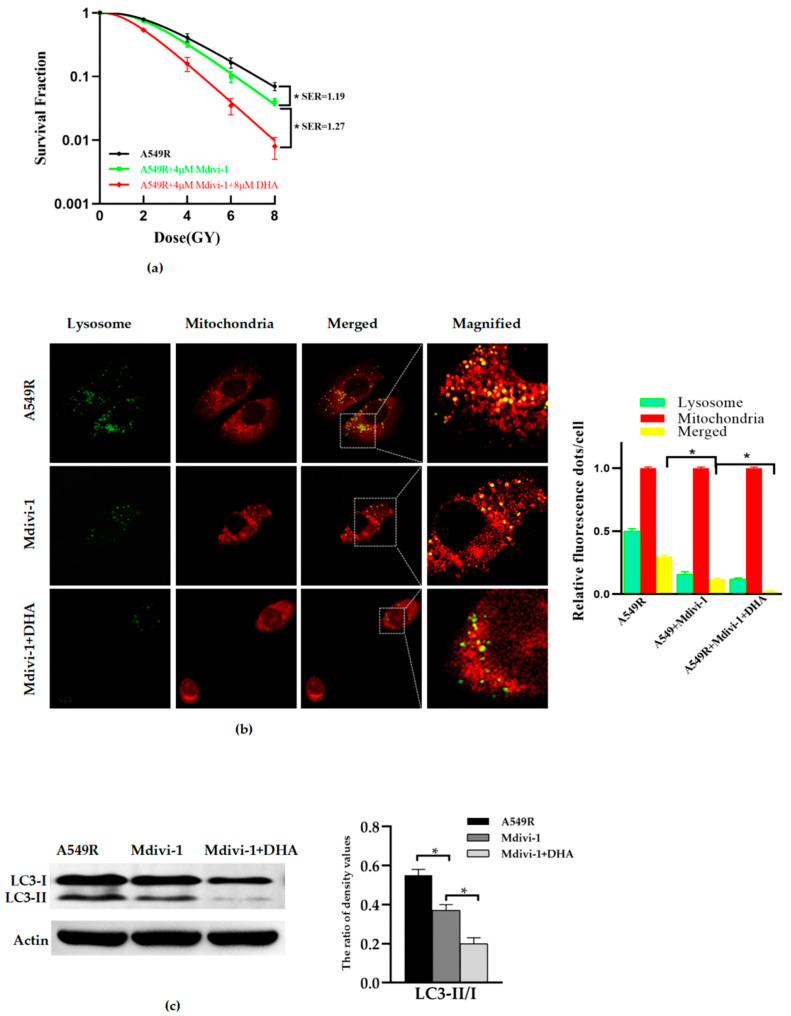
Inhibition of mitophagy reduces the radioresistance of A549R cells. (**a**) The survival fractions of A549R cells treated with Mdivi-1 (4 μM, 24 h) and/or DHA (0–8 μM, 24 h) were fitted by the multitarget–single hitting model according to a clonogenic assay. (**b**) Representative fluorescence images of A549R cells treated with Mdivi-1 (4 μM, 24 h) and/or DHA (8 μM, 24 h) treated with Mito-Tracker (Red) and Lyso-Tracker (Green) (×40) (**Left**). Relative fluorescence spot numbers per cell. A total of 100 cells were counted for each analysis (**right**). (**c**) Western blot assay of LC3-I, LC3-II, and actin proteins and the relative ratio of LC3-II/LC3-I in A549R cells treated with Mdivi-1 (4 μM, 24 h) and/or DHA (8 μM, 24 h). * *p* < 0.05 between the indicated groups.

Based on previous research, the mitochondrial division inhibitor 1 (Mdivi-1) was extensively studied and found to reduce dynamin-related protein 1 (Drp1) levels and excessive mitochondrial fission, and can inhibit mitophagy through mitochondrial-targeting decreasing PINK1, PARK2 and LC3II levels, both in vivo and in vitro [11,13,14,15]. In order to clarify the effect of mitophagy on the radioresistance of A549R cells, Mdivi-1 was used in this study. The radiation sensitivity and mitophagy of A549R cells treated with Mdivi-1 or Mdivi-1 combined with DHA were identified. The results showed that Mdivi-1 (4 μM, 24 h) can significantly reduce the radioresistance (Figure 3a) and mitophagy (Figure 3b,c) of A549R cells. Mdivi-1 (4 μM, 24 h) combined with DHA (8 μM, 24 h) further reduced the mitophagy and radioresistance of A549 cells, compared to Mdivi-1 alone. Putting these together, we can conclude that DHA may reduce the radioresistance of lung cancer A549R cells by inhibiting mitophagy.

### 3.4. CIRBP Identified as a Potential Target of DHA-Reducing Mitophagy and Radioresistance

**Figure 4:**
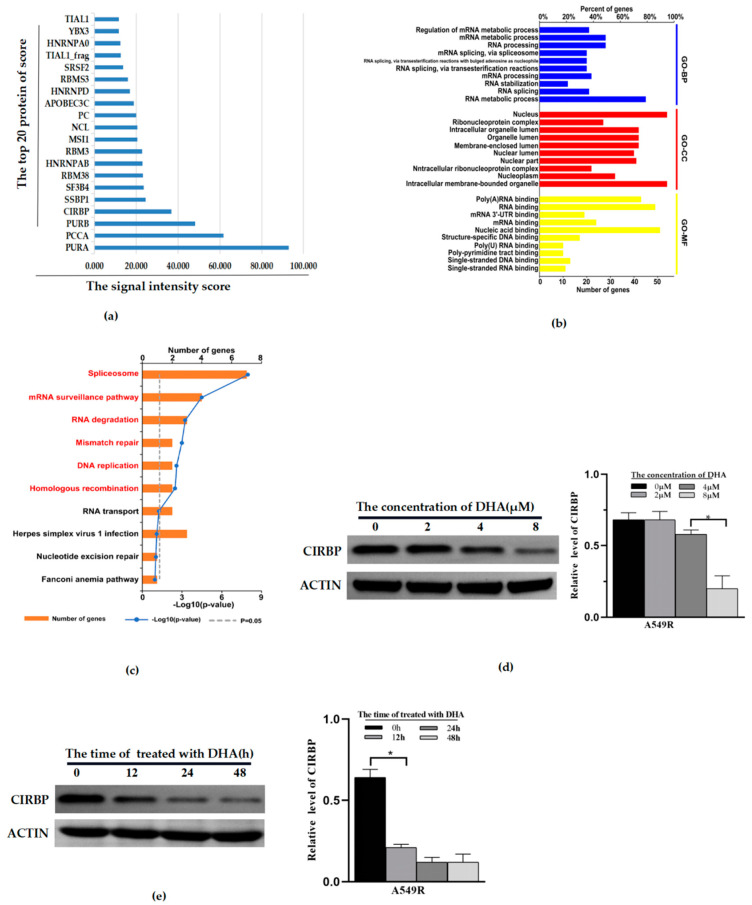
CIRBP is a potential target gene of DHA-reducing mitophagy and radioresistance. (**a**) Proteins with the top 20 signal intensity scores, according to the protein chip. (**b**) GO analysis of the cellular components, biological processes, and molecular functions involved in differential proteins. (**c**) KEGG analysis of differential proteins, according to the protein chip. (**d**) The protein expression of CIRBP in A549R cells treated with DHA (0–8 μM, 24 h). (**e**) The protein expression of CIRBP in A549R cells treated with DHA (0–48 h). * *p* < 0.05 between the indicated groups.

In order to explore the mechanism of DHA regulating mitophagy and then affecting radioresistance, a protein chip assay was used. All proteins whose two reset points met Z-score ≥ 2.8 were screened and sorted according to the site correction signal intensity score. Then, the duplicate protein sites were removed, and the proteins of the top 20 scores were obtained (Figure 4a). Next, we queried the structure, expression, and molecular function of these proteins in the PubMed database. It was found that only CIRBP, NCL, and HNRNPD are related to both mitochondrial function and autophagy, as well as anti-tumor biological activity. In addition, we used bioinformatics methods (GO and KEGG) to analyze the cellular components, biological processes, and molecular functions involved in differential proteins. It was found that these proteins are involved in various cellular biological processes, including regulation of the mRNA metabolic process and RNA binding (Figure 4b). In particular, CIRBP is mainly involved in the mRNA metabolic process, mRNA stabilization, regulation of gene expression and so on. CIRBP must play a vital role in cells’ biological characteristics. Meanwhile, CIRBP achieved the highest signal score; therefore, we took CIRBP as the potential target for DHA-regulating mitophagy for further research. We detected the protein expression of CIRBP in the A549R cell line. The results showed that the expression of CIRBP was significantly time-dependent (Figure 4d). After treatment with DHA (8 μM, 24 h), the expression of CIRBP in A549R was reduced significantly (Figure 4c).

### 3.5. Knockdown of CIRBP Inhibited the Mitophagy and Radioresistance of A549R

**Figure 5:**
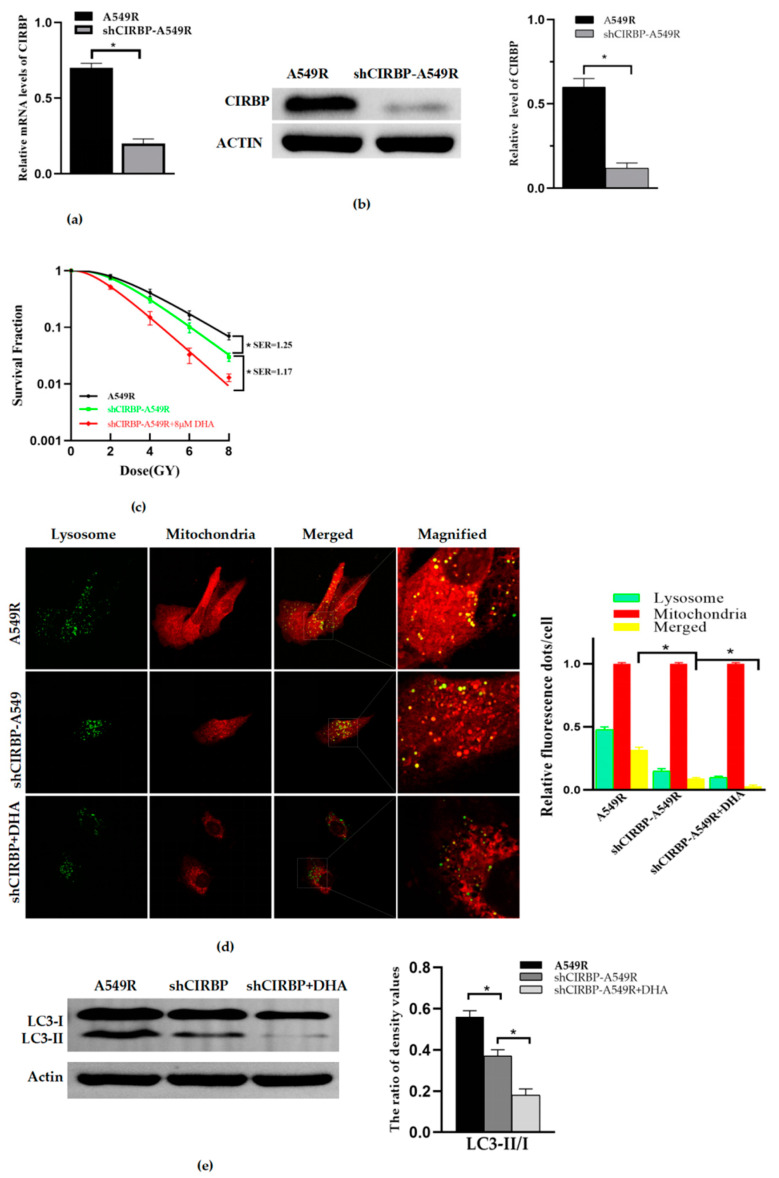
Knockdown of CIRBP inhibits the mitophagy and radioresistance of A549R. (**a**) The relative RNA level of CIRBP with or without CIRBP knockdown in A549R cells. (**b**) The relative protein level of CIRBP with or without CIRBP knockdown in A549R cells. (**c**) The survival fractions of A549R cells and shCIRBP–A549R with or without DHA (8 μM, 24 h) were fitted by the multitarget–single hitting model according to a clonogenic assay. (**d**) Representative fluorescence images of A549R cells and shCIRBP–A549R cells with or without DHA (8 μM, 24 h) treated with Mito-Tracker (Red) and Lyso-Tracker (Green) (×40) (**Left**). Relative fluorescence spot numbers per cell. A total of 100 cells were counted for each analysis (**right**). (**e**) Western blot assay of LC3-I, LC3-II, and actin proteins and the relative ratio of LC3-II/LC3-I in A549R cells and shCIRBP-A549R cells with or without DHA (8 μM, 24 h). * *p* < 0.05 between the indicated groups.

In order to clarify the effect of CIRBP on DHA-reducing radioresistance of A549R, we transfected A549R with a lentivirus vector to construct a stable cells line with low expression of CIRBP, shCIRBP–A549R. RT-PCR (Figure 5a) and Western blot (Figure 5b) showed that the RNA and protein expression of CIRBP was inhibited in shCIRBP–A549R. The results showed that the RNA and protein levels of shCIRBP–A549R cells decreased 71.4% and 80%, respectively, compared with the A549R cells. Then, combined with or without DHA treatment, we found that knockdown of CIRBP inhibited the mitophagy and radioresistance of A549R (Figure 5c); however, combined with DHA, it could further reduce the mitophagy and radioresistance of A549R (Figure 5d,e).

### 3.6. Knockdown of CIRBP Inhibited the PINK1/Parkin Pathway

**Figure 6:**
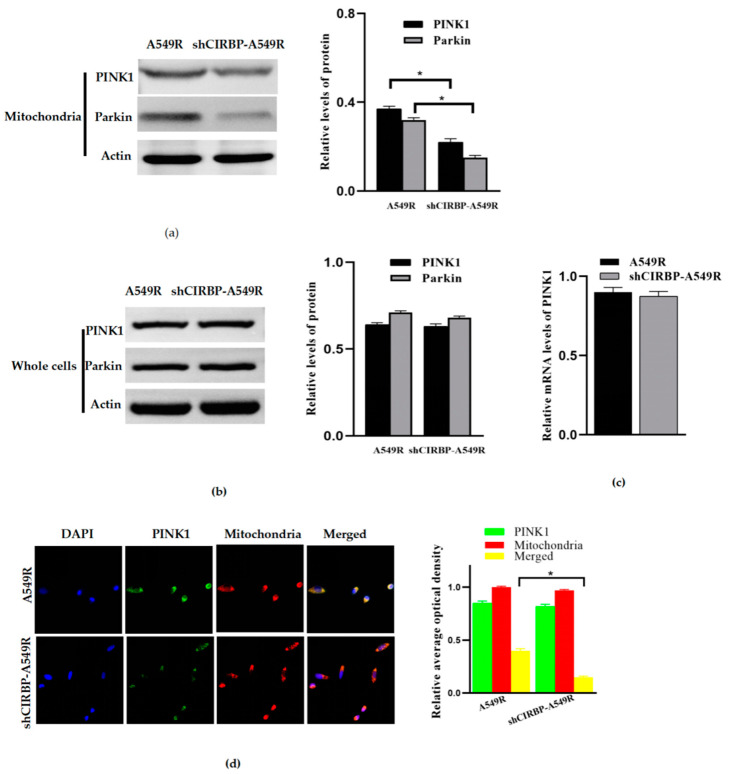
Knockdown of CIRBP inhibits the PINK1/Parkin pathway. (**a**) The influence of the knockdown of CIRBP on the protein expression of PINK1 and Parkin in the mitochondria of A549R. (**b**) The influence of the knockdown of CIRBP on the proteins PINK1 and Parkin in the whole cells of A549R. (**c**) The influence of the knockdown of CIRBP on PINK1 mRNA in A549R cells. (**d**) The influence of the knockdown of CIRBP on the accumulation of PINK1 in mitochondria (×10). * *p* < 0.05 between the indicated groups.

In order to explore the molecular mechanism of CIRBP-regulating mitophagy, we examined the effect of the knockdown of CIRBP on the expression of mitophagy-related proteins PINK1 and Parkin. The results showed that the protein expression of PINK1 and Parkin in mitochondria decreased with CIRBP knockdown (Figure 6a). However, at the whole cell level, we found that the protein of PINK1 did not decline significantly (Figure 6b), and RT-PCR showed that PINK1 mRNA also did not decrease significantly (Figure 6c). Confocal imaging showed that the knockdown of CIRBP decreased the accumulation of PINK1 in mitochondria (Figure 6d). Together, it is reasonable to speculate that the knockdown of CIRBP inhibits the translocation of PINK1 from the cytosol to the mitochondria or promotes the degradation of PINK1 in mitochondria, but does not influence the transcription level of PINK1.

## 4. Discussion

Lung cancer is the most common malignant tumor and the leading cause of cancer-related death. The progress of technology has resulted in the radiotherapy of tumors achieving more accurate targets and reducing the irradiation to the surrounding normal tissues. This has expanded the indications of radiotherapy for lung cancer and has improved survival and reduced toxicity [1]. However, in some patients, the resistance of cancer to treatment results in residual, further recurrence, and metastasis [2]. Conventionally, lung cancer is treated by fraction radiotherapy, generally 2 Gy a day, five times a week, with a total dose of 60–70 Gy [2]. In addition, fractional radiation has been found to produce radioresistance. For example, the radioresistance of nasopharyngeal carcinoma CNE1 cells can be elevated by long-term fractional X-ray irradiation with a total dose of 60 Gy [10]. Fraction radiotherapy induces epithelial–mesenchymal transition and radioresistance in a cellular manner [16]. Fraction irradiation enhances invasion and migration in A549 cells [16,17]. Therefore, in this study, we also tried to construct radiation-resistant lung cancer cells with fractional radiation (similar to conventional fraction radiotherapy). The results showed that lung cancer A549 cells had stronger radioresistance and higher mitophagy after suffering fractional radiation (2 Gy per day, continuous radiation for 20 days), compared to A549 cells without exposed radiation.

Autophagy is a double-edged sword, which has the dual effects of cell protection and cytotoxicity. The effect of autophagy depends on the type of cells and the stress state [18]. Autophagy is very important for tumor cells to survive under stress, which is related to tumor resistance to chemotherapy and radiotherapy. Mitophagy contributes to the clearance and renewal of damaged mitochondria, so as to protect cells from stress [19]. We found that the level of mitophagy was elevated in radioresistant A549R cells, and inhibition of mitophagy can increase the radiosensitivity of A549R cells, which indicates that mitophagy plays a protective role and that is why such a high mitophagy level occurs after suffering from repeated irradiation. A high level of mitophagy results in a minimum amount of damaged mitochondria retained in A549R cells. In other words, in A549R cells, the rapid renewal of mitochondria leads to the presence of a large number of newly generated mitochondria, which have not been stimulated by radiation, and therefore, they produce low levels of ROS, which can reasonably explain why the ROS level in A549R is lower than in A549 cells.

DHA is a synthetic derivative of artemisinin; an increasing number of studies report that DHA possesses anticancer activity on many types of cancer, both in vitro and in vivo, as well as enhances the efficacy of chemotherapy, target therapy, and radiotherapy [20]. However, the mechanisms of DHA on different tumors differ in various ways. DHA can increase the inhibition of sorafenib on liver cancer [21], increase the effect of gefitinib on lung cancer by regulating the cell cycle [22], and increase the sensitivity of lung cancer and colorectal cancer to radiotherapy by activating of anti-oxidant Keap1/Nrf2 pathway [23]. In addition, DHA can also regulate autophagy. DHA can promote autophagy and increase the inhibition of rapamycin, doxorubicin, and temozolomide on breast cancer [24], lung cancer [25] and glioma [26], respectively. However, there are few studies on the effect of DHA on mitophagy. Our research showed that DHA can reduce the mitophagy and radioresistance of A549R cells. The inhibition of mitophagy can reduce the radiation resistance of lung cancer. The treatment of an inhibitor Mdivi-1 combined with DHA can significantly reduce the mitophagy and radiation resistance further. Therefore, we believe that DHA may reduce the radioresistance of lung cancer by inhibiting mitophagy. However, in our study, we failed to detect the effect of DHA on A549 cells and lower concentration levels of DHA on A549R cells. We speculate that the concentration is a key factor affecting DHA’s efficacy. In addition, A549R cells which had undergone continuous radiation were in a state of heightened stress, perhaps making them more sensitive to DHA. Therefore, DHA (8 μM) could inhibit the mitophagy and radioresistance of A549R cells, but not of A549 cells.

The types of post-radiation cell death include apoptosis, autophagy, necrosis, aging and mitotic disaster and so on. In this study, we have only researched the cells death associated with mitophagy (a selective autophagy). We have found that the high level mitophagy and low level ROS, which means lower DNA damage, may be the causes of radioresistance. When treated with DHA, the mitophagy was inhibited, and produced more ROS, which resulted in higher radiosensitivity and increased cell death. The mechanisms of cell death are complex and it should coexist in many ways simultaneously. Other mechanisms of death, including apoptosis, will be the subject of our next study.

Protein chips are a technology based on high-throughput protein function analysis. At present, protein chips are widely used in the screening of targets of drugs, among others. According to our preset conditions (double reset point scores greater than 2.8), we obtained 62 potential targets of DHA. We queried the molecular functions of the top 20 proteins in the PubMed database. Combined with bioinformatics analysis, CIRBP is supposed to be the most likely potential target for DHA-regulating mitophagy.

CIRBP is a cold-shock protein comprising an RNA-binding motif that is induced by several stressors, such as cold shock, UV radiation, nutrient deprivation, ROS, and hypoxia [27]. The crucial function of CIRBP has been reported in various human diseases, including cancers. As a tumor suppressor, CIRBP decreases the proliferation in rectal carcinoma cells and ovarian cancer cells by binds the 5′ and 3′-UTRs of mRNAs, as well as poly U sequences at the 3′-ends to regulate the polyadenylation and 3′-end cleavage, and increases the translational efficiency of DNA damage response genes ATR, Trx-1, and RPA2 [28]. As a tumor promoter, CIRBP can promote proliferation and reduce apoptosis in breast cancer [29], prostate cancer [30], and lung carcinoma cells (A549) [31] by target mRNA. Importantly, data show that CIRBP induces ROS and mitochondrial DNA fragmentation and regulates macrophage autophagy through TLR4 signaling [32]. Therefore, we speculate that CIRBP may be closely related to the mitophagy of A549R cells. Our findings are consistent with this hypothesis. The knockdown of CIRBP reduced the mitophagy and radioresistance of A549R and increased the inhibition of DHA on the radioresistance of A549R. Together, this evidence demonstrates that DHA reduces irradiation-induced mitophagy and radioresistance in lung cancer A549 cells via CIRBP inhibition.

The PINK1/Parkin pathway, closely related to mitophagy, is one of the most well-characterized mitophagy pathways, but the mechanism of PINK1-dependent mitophagy in radioresistance remains elusive [9,10,11,33]. In order to explore the molecular mechanism of the knockdown of CIRBP-inhibiting mitophagy, we detected the protein expression of PINK1 and Parkin. The results showed that the knockdown of CIRBP inhibited the protein expression of PINK1 and Parkin in mitochondria, but failed to influence the expression of RNA and the whole cell protein of PINK1. In other words, CIRBP promotes mitophagy by promoting the translocation of PINK1 from the cytoplasm to mitochondria or degradation in mitochondria, rather than regulating the transcription of PINK1 RNA. Future studies should be aimed at elucidating the molecular mechanism of DHA-inhibiting CIRBP and CIRBP-regulating PINK1 translocation.

In conclusion, we elucidated the function of DHA in inhibiting mitophagy and reducing the radioresistance of A549R cells, and identified that CIRBP may be the key target of DHA. DHA may be a useful radiation sensitizer, and prospectively, CIRBP may be a potential therapeutic target for NSCLC.

## Data Availability

Data available in a publicly accessible repository.
